# More patient-centered care, better healthcare: the association between patient-centered care and healthcare outcomes in inpatients

**DOI:** 10.3389/fpubh.2023.1148277

**Published:** 2023-10-19

**Authors:** Chenhao Yu, Yun Xian, Tiantian Jing, Mayangzong Bai, Xueyuan Li, Jiahui Li, Huigang Liang, Guangjun Yu, Zhiruo Zhang

**Affiliations:** ^1^School of Public Health, Shanghai Jiao Tong University School of Medicine, Shanghai, China; ^2^Health Commission of Shanghai Huangpu, Shanghai, China; ^3^Shanghai Municipal Center for Disease Control and Prevention, Shanghai, China; ^4^Department of Business and Information Technology, Fogelman College of Business and Economics, University of Memphis, Memphis, TN, United States; ^5^School of Medicine, The Chinese University of Hong Kong, Shenzhen, Guangdong, China

**Keywords:** patient-centered care, physician-induced demand, charge and fees, healthcare expense, healthcare insurance sustainability

## Abstract

**Objective:**

The objective of this study is to explore the association between patient-centered care (PCC) and inpatient healthcare outcomes, including self-reported physical and mental health status, subjective necessity of hospitalization, and physician-induced demand behaviors.

**Methods:**

A cross-sectional survey was conducted to assess patient-centered care among inpatients in comprehensive hospitals through QR codes after discharge from September 2021 to December 2021 and had 5,222 respondents in Jiayuguan, Gansu. The questionnaire included a translated 6-item version of the PCC questionnaire, physician-induced behaviors, and patients' sociodemographic characteristics including gender, household registration, age, and income. Logistic regression analyses were conducted to assess whether PCC promoted self-reported health, the subjective necessity of hospitalization, and decreased physician-induced demand. The interactions between PCC and household registration were implemented to assess the effect of the difference between adequate and inadequate healthcare resources.

**Results:**

PCC promoted the patient's self-reported physical (OR = 4.154, *p* < 0.001) and mental health (OR = 5.642, *p* < 0.001) and subjective necessity of hospitalization (OR = 6.160, *p* < 0.001). Meanwhile, PCC reduced physician-induced demand in advising to buy medicines outside (OR = 0.415, *p* < 0.001), paying at the outpatient clinic (OR =0.349, *p* < 0.001), issuing unnecessary or repeated prescriptions and medical tests (OR = 0.320, *p* < 0.001), and requiring discharge and readmitting (OR = 0.389, *p* < 0.001).

**Conclusion:**

By improving health outcomes for inpatients and reducing the risk of physician-induced demand, PCC can benefit both patients and health insurance systems. Therefore, PCC should be implemented in healthcare settings.

## Introduction

The WHO advocates for patient-centered care (PCC) in healthcare (1), taking into account patients' perspectives and psychological and interpersonal effects during treatment (2). Despite different definitions of PCC (3), the core of achieving PCC is to inform and involve patients in healthcare (4). The involvement of patients shifts the focus of healthcare from the disease to the patient, which promotes the wellbeing of the patients (2).

PCC has been shown to improve healthcare outcomes for a variety of chronic diseases, including depression and anxiety disorder (5–7), cardiovascular risk management (8–11), diabetes (12–15), and addictive behavior (16). The benefits of PCC include improved patient engagement, which can lead to better treatment outcomes and lower costs. PCC also allows for the integration of multidisciplinary team engagement and nutrient management, which can play a greater role in chronic disease management (17–20).

However, some studies have found that PCC does not have a significant impact on health status. For example, Spall et al. (21) found that PCC treatment did not decrease the readmission rate of heart failure patients. Ma et al. (22) also found that PCC improved the self-efficacy of patients diagnosed with diabetes, but the health status remained the same as the control group.

These findings suggest that the impact of PCC on health status may vary depending on the patient population. For example, PCC may be more effective for patients with psychological conditions, such as anxiety or depression. It is also possible that the heterogeneity of patients in randomized controlled trials (RCTs) has limited the ability to detect a significant effect of PCC on health status. For example, RCTs may exclude patients who are most likely to benefit from PCC, such as the older adult (21, 23).

Overall, the evidence on the impact of PCC on health status is mixed. Therefore, it is still essential to study the association between PCC and health status, as well as the subjective necessity of healthcare utilization in the population. Considering that the role of PCC is realized through all aspects of patients' daily lives, we focus on patients' self-reported health status and subjective necessity of hospitalization. These indicators are not only convenient to collect but also they are reliable in predicting patients' behaviors (24, 25).

Under the World Trade Organization (WTO) framework, the social cost of healthcare must also be considered (26). Some studies of PCC in populations have focused on the cost of treatment, which has led to contradictory findings about the financial effects of PCC.

For example, Liang et al. (23) reviewed PCC in oncology care and found that PCC reduced the utilization of inpatient care and cost. However, the overall positive effects were not statistically significant. Kohler et al. (27) also found that PCC promoted healthcare utilization in primary care and emergency care. David et al. (28) proposed that the contradictory findings may be due to the heterogeneity of patient populations. They found that PCC had differential effects on different patient populations.

From the perspective of information asymmetry between patients and physicians (29), the patient-centered approach can help to reduce information asymmetry between patients and physicians, which can lead to more effective healthcare.

In a patient-centered approach, the physician provides the patient with more information about their treatment plan and disease. This helps to fill the information gap between the patient and physician (2), which can lead to better decision-making. Hence, a patient-centered approach can help to reduce physician-induced demand and patients' self-interested behaviors. Physician-induced demand occurs when a physician orders unnecessary tests or procedures because they believe the patient will want them.

According to the regulations of Chinese healthcare insurance, we focus on four potential healthcare insurance violation behaviors, i.e., advising to buy medicines outside, paying at the outpatient clinic, issuing unnecessary or repeating prescriptions and medical tests, and requiring discharge and readmitting. We focus on these behaviors due to two main reasons. First, physicians may have a financial incentive to do so. They may have an interesting relationship with a specific pharmacy, and they may receive kickbacks or other benefits for referring patients to that pharmacy. Second, physicians may be motivated by performance metrics. The proportion of medicines that are prescribed by a physician is often used as a key performance indicator (KPI). If a physician wants to improve the KPI, they may be tempted to advise patients to buy medicines outside of the hospital, even if it is not in the best interests of the patient. Meanwhile, the average length of hospital stay is also a KPI; hence the physician may be tempted to advise patients to pay at the outpatient clinic and require discharge and readmitting.

Focusing on the expense caused by physician-induced demand has two advantages over direct comparison of expense. On one hand, healthcare expenses can vary due to the patient's physical condition and diagnosis techniques, which makes it difficult to compare expenses between different patients directly (23). Moreover, the expense caused by physician-induced demand can be identified by the specific behaviors prohibited by regulation, which avoids the need for a direct comparison of expenses. On the other hand, reducing physician-induced demand is a more critical issue in the sustainability of healthcare insurance (30). Therefore, we focus on whether PCC can reduce the expense caused by healthcare insurance fraud.

In summary, this study explored whether PCC could promote patients' self-reported health status and subjective necessity of hospitalization and reduce physician-induced demand.

## Methods

### Study design and participants

The study was conducted in the secondary and tertiary hospitals of Jiayuguan, a prefecture-level city in northwestern Gansu province in China, with 312,000 residents. To keep the representativeness of the sample, this study includes the inpatients of the tertiary (tertiary referral) and secondary (regional or district) comprehensive hospitals in Jiayuguan.

This study distributed questionnaires among inpatients (*n* = 5,222) by posting QR codes. The survey of the study was implemented from September 2021 to December 2021. The incomplete questionnaires were removed (*n* = 23). Research ethics approval was obtained from Shanghai Jiao Tong University School of Medicine (protocol code STUPN-202203). All participants were given informed consent before the study began.

### Description of variables

**Figure 1:**
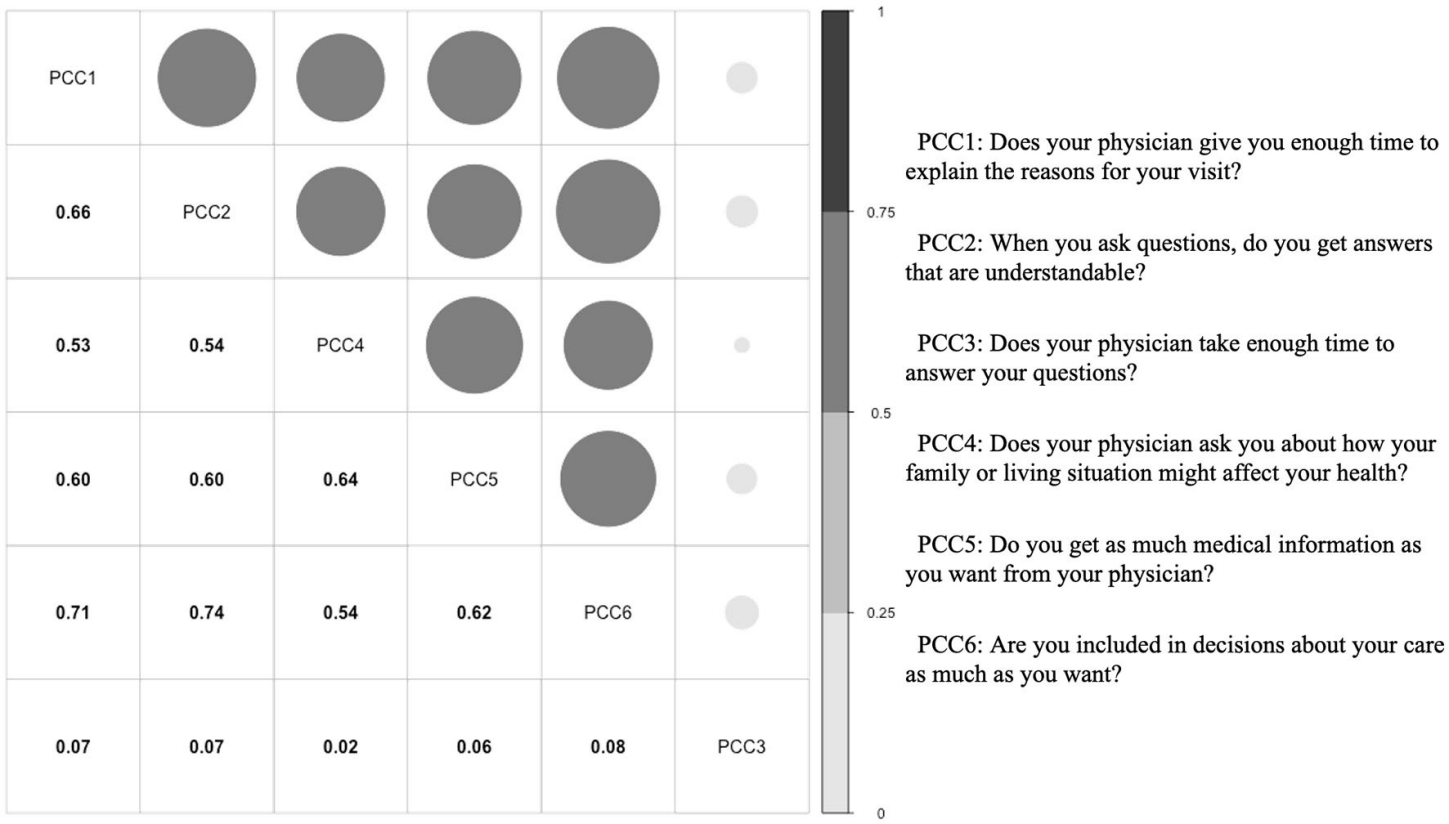
The correlation among PCC items.

A translated version of the 6-item patient-centered care scale developed by Keating et al. (31) was used to assess patient-centered care. The scale uses a five-point Likert scale, ranging from 1 (never) to 5 (always), to assess the degree to which patients agree with statements about their care. One item, “Does your physician take enough time to answer your questions?” was deleted from the scale for two reasons. First, nurses spend more time communicating directly with hospitalized patients, so the communication between patients and physicians may compete with the communication between patients and nurses (32, 33). Second, Cronbach's alpha of the scale increased from 0.53 to 0.88 after this item was deleted, and the item had a very low correlation with the other items (detailed in Figure 1). The average score was used to reflect the level of PCC perceived by the patient, with higher scores representing higher PCC. The 6-item scale was also implemented in the supplementary document to improve the robustness of the study (see details in Supplementary Tables 1, 2).

The self-reported health status was assessed using three questions: “How would you rate your current physical/mental health?” and “How much do you think your hospitalization is necessary?” The questions were answered on a scale of 1 (absolutely disagree/very bad) to 5 (absolutely agree/very good). The participants were inpatients, and the questionnaires were collected when they were filling discharge procedures. Therefore, the self-reported health status can be used to measure the effect of healthcare.

To assess the physician-induced demand (34), we have collected information about whether the physician performed the following behaviors which had been prohibited by regulations, i.e., advising to buy medicines outside, paying at the outpatient clinic, issuing unnecessary or repeated prescriptions and medical tests, and requiring discharge and readmitting.

The control variables include the types of household registration (0 = agricultural household registration, 1 = non-agricultural household registration) that would not only indirectly affect the occupation or work sector but also affect the type of medical insurance for inpatients, gender (0 = male, 1 = female), age, education status (0 = did (or not) finish primary school, 1 = junior high school, 2 = high school or secondary school, 3 = college, 4 = undergraduate, 5 = master degree, and 6 = doctoral degree), marital status (0 = unmarried, 1 = married, 2 = divorced, and 3 = widowed), and average yearly income (0 = lower than 2800 Yuan, 1 = 2,801–10,000 Yuan, 2 = 10,001–30,000 Yuan, 3 = 30,001–100,000 Yuan, 4 = 100,001–300,000 Yuan, and 5 = more than 300,001 Yuan).

### Analytical scheme

Multiple logistic regressions were implemented to estimate the effect of PCC. We used health status and perceived necessity to healthcare utilization as binary variables, with a value of 1 indicating a response of >3 (35). To account for the group characteristics of different hospitals during data collection, we controlled for the random effect from different hospitals and used a robust cluster estimator (36) grouping with the hospital. All statistical analyses were performed in R 4.1.1 with the packages psych 2.2.9 and RMS 6.4-0.

## Results

### Descriptive statistics

The socioeconomic status of participants is presented in Table 1. The majority of participants were female (52.4%) and urban residents (76.1%). The mean age was 48.76 years old. Most participants had an annual income of <100,000 yuan. Only 6.7% of participants did not have government-provided healthcare insurance.

**Table 1:** Descriptive statistics (*N* = 5,199).

**Variable**	**Mean**	**Std. Dev**.	**Min**	**Max**
PCC1	4.796	0.478	1	5
PCC2	4.824	0.451	1	5
PCC3	3.627	1.686	1	5
PCC4	4.741	0.572	1	5
PCC5	4.766	0.524	1	5
PCC6	4.822	0.45	1	5
Mean of PCC (5 items)	4.79	0.411	1	5
Mean of PCC (6 items)	4.596	0.458	1.667	5
Age	48.762	14.808	18	97
	**Percentage (%)**	**Std. Dev**.	**Min**	**Max**
Physical health status (PHS)	76.3%	0.425	0	1
Mental health status (MHS)	90.0%	0.3	0	1
Patients' subjective necessity of hospitalization (PSN)	99.7%	0.052	0	1
Advising to buy medicines outside (ABM)	5.80%	0.233	0	1
Paying at the outpatient clinic (PO)	6.10%	0.239	0	1
Issuing unnecessary or repeated prescriptions and medical tests (IU)	2.00%	0.14	0	1
Requiring discharge and readmitting (RDR)	1.6%	0.126	0	1
Household registration (0 = Agricultural)	76.1%	0.426	0	1
Income (Yuan)	.	.	.	.
< 2,800	14.9%	0.356	0	1
2,801 ~ 10,000	29.1%	0.454	0	1
10,001 ~ 30,000	22.2%	0.416	0	1
30,001 ~ 100,000	28.4%	0.451	0	1
100,001 ~ 300,000	5.0%	0.218	0	1
More than 300,000	0.40%	0.063	0	1
Gender (0 = Male)	47.6%	0.499	0	1
Marital status (0 = Unmarried)	93.6%	0.254	0	1
Education	.	.	.	.
< = Primary school	10.1%	0.301	0	1
Junior high school	20.4%	0.403	0	1
High or secondary school	29.5%	0.456	0	1
College	24.9%	0.432	0	1
Undergraduate	14.4%	0.351	0	1
Master degree	0.5%	0.073	0	1
Doctoral degree	0.1%	0.037	0	1
Healthcare insurance (0 = No)	93.2%	0.251	0	1

### The association between patient-centered care and health status

Table 2 presents the association between PCC and health status. Based on the identification, PCC played as a protective factor for self-reported physical health status (OR = 4.154, *p* < 0.001) and self-reported mental health status (OR =5.642, *p* < 0.001). PCC also promotes the patient's subjective necessity of hospitalization (OR =6.160, *p* < 0.001).

**Table 2:** The association between PCC and health status and subjective necessity of hospitalization.

	**Dependent variable**					
	**PHS**	**MHS**	**PSN**	**PHS**	**MHS**	**PSN**
	**(1)**	**(2)**	**(3)**	**(4)**	**(5)**	**(6)**
PCC	4.154^***^	5.642^***^	6.160^***^	7.169^***^	10.552^***^	5.965^***^
	(0.064)	(0.037)	(0.118)	(0.052)	(0.075)	(0.030)
Age	0.978^***^	0.986^***^	0.968^***^	0.978^***^	0.986^***^	0.968^***^
	(0.007)	(0.006)	(0.024)	(0.007)	(0.006)	(0.023)
Household registration	0.733^***^	0.698^***^	1.704^***^	19.452^***^	26.081^***^	1.367^***^
	(0.049)	(0.080)	(0.654)	(0.673)	(0.328)	(0.238)
Income	1.022^***^	1.206^***^	0.864^***^	1.024^***^	1.208^***^	0.861^***^
	(0.060)	(0.011)	(0.144)	(0.059)	(0.012)	(0.154)
Gender	0.968^***^	1.030^***^	0.929^***^	0.974^***^	1.043^***^	0.928^***^
	(0.135)	(0.053)	(0.221)	(0.132)	(0.050)	(0.218)
Marital status	0.826^**^	0.809^**^	3.855^***^	0.819^**^	0.789^**^	3.834^***^
	(0.386)	(0.316)	(0.238)	(0.386)	(0.320)	(0.222)
Education	0.962^***^	1.083^***^	0.909^***^	0.962^***^	1.082^***^	0.914^***^
	(0.014)	(0.028)	(0.347)	(0.014)	(0.029)	(0.333)
Health insurance	1.624^***^	1.309^***^	0.743	1.715^***^	1.410^***^	0.727
	(0.205)	(0.240)	(0.597)	(0.213)	(0.262)	(0.656)
PCC ^*^ Residence				0.497^***^	0.449^***^	1.057^***^
				(0.137)	(0.084)	(0.183)
Constant	0.018	0.005	0.137	0.001	0.0003	0.154
	(0.384)	(0.227)	(1.083)	(0.221)	(0.437)	(1.424)
Observations	5,199	5,199	5,199	5,199	5,199	5,199
Adj-R^2^	0.161	0.169	0.200	0.165	0.173	0.200

The ^*^p < 0.01, ^**^p < 0.01, ^***^p < 0.01. The values in parentheses are the clustered robust standard errors. PHS, Physical health status; MHS, Mental health status; PSN, Patients' subjective necessity of hospitalization; PCC, Patient-centered care.

**Figure 2:**
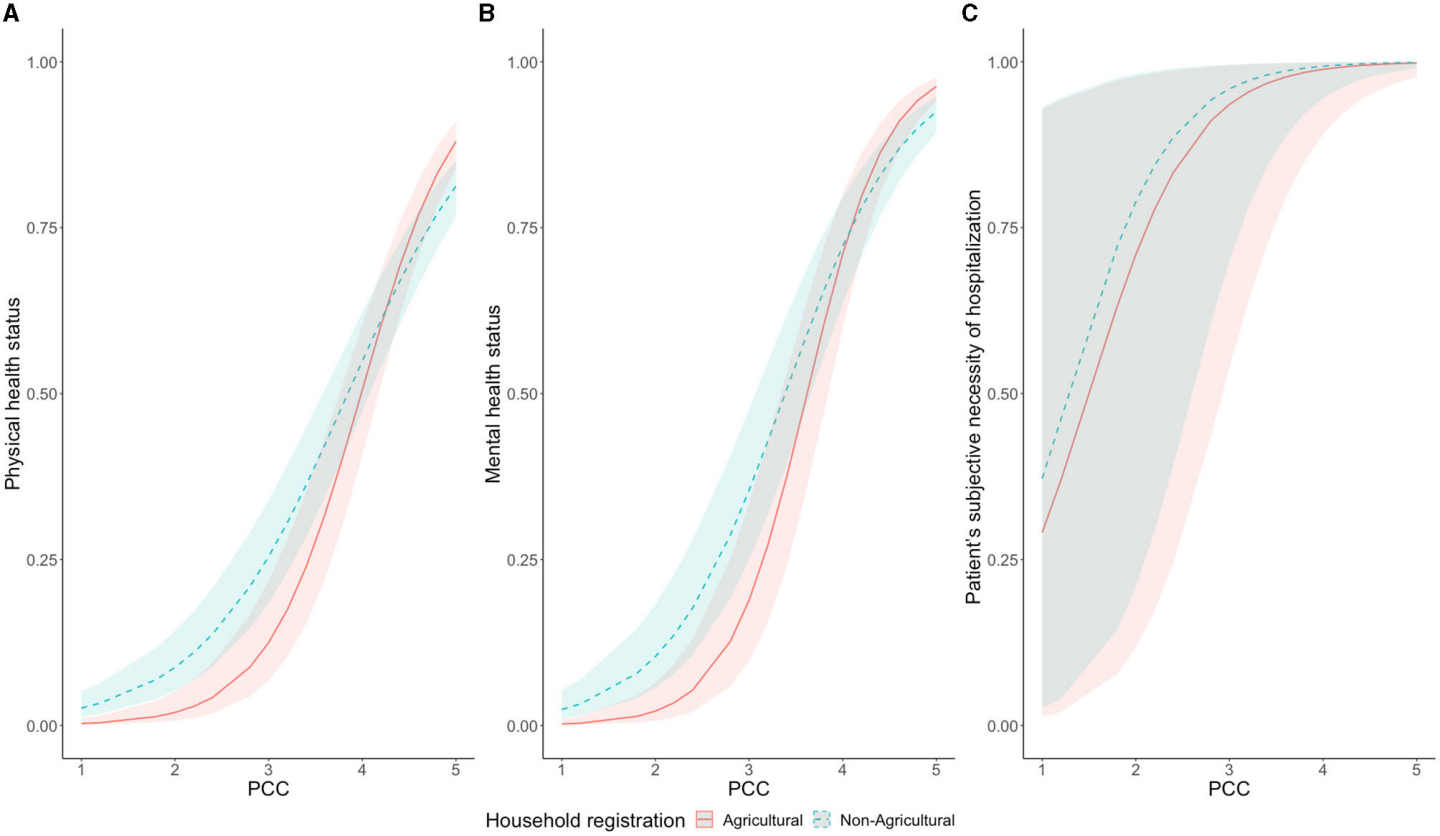
The interaction relationship between PCC and PHS **(A)** and MHS **(B)** and PSN **(C)**. PHS, Physical health status; MHS, Mental health status; PSN, Patients' subjective necessity of hospitalization; PCC, Patient-centered care.

We found that household registration status had different effects on the three outcomes. This led us to estimate whether household registration status interacted with patient-centered care (PCC). As shown in Figure 2 and columns (4–6) of Table 2, we found that when the mean score of PCC was above 4, the self-reported physical and mental health of urban residents was lower than those of agricultural household registration individuals.

### The association between patient-centered care and physician-induced demand

Table 3 shows the association between PCC and physician-induced demand. A higher PCC score was associated with a lower probability of physician-induced demand. Notably, the role of PCC was close and sufficient in all four healthcare insurance violations. From the perspective of comparing the scale of coefficients, PCC could prevent physicians from issuing unnecessary or repeated prescriptions and medical tests (OR = 0.320, *p* < 0.001), which was most relevant to physician self-interest. We also found that marital status could change physician behavior. Married patients had a higher opportunity to be advised to buy medicines outside (OR = 1.385, *p* < 0.001) or to pay at the outpatient clinic (OR = 3.221, *p* < 0.001), which were usually done by the spouse of the patient.

**Table 3:** The association between PCC and physician-induced demand.

	**Dependent variable**			
	**ABM**	**PO**	**IU**	**RDR**
	**(1)**	**(2)**	**(3)**	**(4)**
PCC	0.415^***^	0.349^***^	0.320^***^	0.389^***^
	(0.050)	(0.064)	(0.064)	(0.077)
Age	0.984^***^	0.958^***^	0.998^***^	1.006^***^
	(0.002)	(0.006)	(0.013)	(0.011)
Household registration	0.973^***^	0.767^***^	0.579	0.555
	(0.187)	(0.277)	(0.376)	(0.385)
Income	0.820^***^	0.909^***^	0.835^***^	0.834^***^
	(0.032)	(0.027)	(0.042)	(0.056)
Gender	0.911^***^	0.699^***^	0.704^***^	0.616^***^
	(0.010)	(0.142)	(0.054)	(0.129)
Marital status	1.385^***^	3.221^***^	0.431^***^	0.336^***^
	(0.204)	(0.196)	(0.156)	(0.068)
Education	0.924^***^	0.963^***^	0.729^***^	0.746^***^
	(0.013)	(0.049)	(0.112)	(0.113)
Health insurance	1.848^***^	1.845^***^	2.308^***^	2.687^***^
	(0.144)	(0.092)	(0.156)	(0.150)
Constant	8.211^***^	18.798^***^	43.927^***^	12.920^***^
	(0.287)	(0.307)	(1.001)	(0.457)
Observations	5,199	5,199	5,199	5,199
Adj-R^2^	0.086	0.139	0.158	0.153

^*^p < 0.01, ^**^p < 0.01, ^***^p < 0.01. The values in parentheses are the clustered robust standard errors. ABM, Advising to buy medicines outside; PO, Paying at the outpatient clinic; IU, Issuing unnecessary or repeated prescriptions and medical tests; RDR, Requiring discharge and readmitting; PCC, Patient-centered care.

## Discussion

This study conducted a large population survey on inpatients in Jiayuguan to explore the association between patient-centered care (PCC), patients' self-reported health status, subjective necessity of hospitalization, and physician-induced demand. The study found that PCC was associated with improved self-reported health status (*p*s < 0.001) and reduced physician-induced demand (*p*s < 0.001). These findings provide new evidence on the controversy about the effect of PCC on health status improvement and asymmetric information between physicians and patients.

### Patient-centered care improving self-reported health status

Unlike previous studies that focused on how to formulate a PCC plan and its effects (37–39), our study provides a macro perspective on the effect of PCC. We found that PCC can generally improve patients' health status and their subjective necessity of hospitalization. Based on the numerous samples from Jiayuguan, we can predict that the association between health status and PCC can be generalized to different types of diseases. Furthermore, our results suggest that PCC can benefit not only chronic diseases but also other diseases by engaging patients in their healthcare plan and providing them with more information and emotional support. As the number of patients with multiple diseases increases due to aging and younger chronic diseases, healthcare orientation needs to shift from disease-centered to patient-centered (40).

PCC is also regarded as a key tool to reduce health disparities (41–43). Considering the large gap in GDP per capita between Jiayuguan and Gansu Province, we tested the different effects of PCC among different socioeconomic status (SES) levels. We used household registration as a moderator to distinguish between high SES and low SES. We found that although PCC can still improve the self-reported physical health and mental health of inpatients, the effect was reduced in the group of non-agricultural household registration. This difference could be caused by the curse effect of education (44) in healthcare, where those with a higher health literacy or better access to medical resources may rank a worse treatment effect during self-report. For inpatients with non-agricultural household registration, due to past prejudices (45), over-care patients are regarded as a kind of palliative care, which leads to the effect of PCC being lower than in inpatients with agricultural household registration.

Considering the epidemiological trend of chronic diseases (46–48) and the current situation of unbalanced medical resources, we believe that providing PCC education in medical education will become an important tool to face future challenges.

### Patient-centered care reducing physician-induced demand

The development of PCC has been criticized by some people for the potential of physicians or hospitals to treat patients as consumers (49), taking advantage of asymmetric information to induce unnecessary medical services (50). However, the evidence on whether PCC reduces physician-induced demand is mixed. We believe that the contradictory findings in previous studies are likely due to two factors (23): (1) the treatment plan for a given patient is highly individualized and (2) the treatment methods available to patients can vary depending on the medical technology available. To address these factors, we have focused on a typical identification of four specific behaviors regulated by medical regulations in China.

From the perspective of healthcare expense, this study provided empirical evidence to support that the PCC plan leads to fewer physician-induced demand behaviors that have already been listed in the regulation and fewer unnecessary expenses. According to the asymmetric information framework, we used the core concept of patient-centered care—involving patients to participate in treatment decisions—to explain the reason why PCC relates to fewer health insurance violations. Moreover, the protective effect of PCC is very sufficient.

In 2021, medical insurance violation funds reached 23.418 billion yuan (51). Incorporating PCC into medical education and practice can improve patient wellbeing and the sustainability of medical insurance. However, the requirement and education of PCC may lead to job burnout (52) for physicians. To solve this, in the short term, the savings of expenses from PCC can be used to expand welfare and job resources for physicians. This can be done through incentive plans that reward physicians for promoting communication and hard work (53). Moreover, in the long term, forming a patient-centered organizational culture (54) and providing psychological safety and perceived organizational support (55) can also be effective methods to promote PCC in practice.

Although the identification of the protective effect of PCC has used Chinese medical regulation, we can still predict that the contradiction of PCC in physician-induced demand and medical expense could be excavated further by eliminating the expense from technological innovation. Our result is consistent with the long-term research on the effect of the Affordable Care Act in the United States (56). Hence, we could predict that PCC would have a generalized effect on reducing physician-induced demand among different countries; however, short-term effects are easily concealed, and its long-term effects need to be identified.

Based on the above discussion, we believe that future research can further proceed from the following three aspects. First, the psychological mechanism for interpreting the reason why PCC would promote mental and physical health status and the patient's subjective necessity of hospitalization would excavate the causation of benefits from PCC. Second, exploring more efficient communication methods based on patient-centered care to promote the feasibility of outpatient is essential. Third, exploring the way to reduce the side-effects of PCC as a job demand from the organizational aspect would promote physicians' wellbeing and make PCC practical.

## Limitations

Although a large survey has been conducted, and we have indicated the association, the mechanism by which PCC reduces asymmetric information between patients and physicians is still worth further exploration.

Additionally, although this study has conducted a large survey of PCC, we cannot guarantee that endogeneity will be eliminated completely. These endogeneities may be caused by the selection of sampling cities and the degree of patient cooperation in the random sampling process. Additionally, whether there are differences between inpatients and outpatients will also affect the generalizability of the results of this study.

## Conclusion

This study provides evidence that patient-centered care (PCC) is an effective and beneficial approach for inpatients in comprehensive hospitals. It shows that PCC can improve patients' physical and mental wellbeing, increase their subjective necessity of hospitalization, and reduce unnecessary or inappropriate medical interventions by physicians. The study also suggests that PCC can have different effects depending on the availability of healthcare resources in different regions. Therefore, PCC should be promoted and tailored to the local context and needs of the patients. These findings call for strengthening PCC research and evidence, underpinning practice, policy, and system transformation. Adopting PCC would provide not only better healthcare but also cost-effective healthcare, which would advance the development of the performance of the healthcare system facing the aging population and tight budget.

## Data availability statement

The raw data supporting the conclusions of this article will be made available by the authors, without undue reservation.

## Ethics statement

The studies involving humans were approved by the Shanghai Jiao Tong University School of Medicine. The studies were conducted in accordance with the local legislation and institutional requirements. The participants provided their written informed consent to participate in this study.

## Author contributions

CY, YX, HL, GY, and ZZ: study design and implementation. CY and HL: methodology. TJ, XL, JL, and MB: data curation. CY and YX: analysis, validation, and writing—original draft preparation. CY, YX, JL, TJ, and MB: discussion. HL, GY, and ZZ: supervision. All authors contributed to the article and approved the submitted version.
