# Supplementary material for: More patient-centered care, better healthcare: the association between patient-centered care and healthcare outcomes in inpatients
Source: Front Public Health. 2023 Oct 19;11:1148277. doi: 10.3389/fpubh.2023.1148277 (PMC10620693; doi:10.3389/fpubh.2023.1148277)
Supplement: Supplementary file 1 [file Table_1.DOCX]

|  | | | | | | |
| --- | --- | --- | --- | --- | --- | --- |
|  | Dependent variable: | | | | | |
|  |  | | | | | |
|  | PHS | MHS | PSN | PHS | MHS | PSN |
|  | (1) | (2) | (3) | (4) | (5) | (6) |
|  | | | | | | |
| PCC-6 | 2.578^***^ | 4.513^***^ | 8.715^***^ | 4.413^***^ | 10.045^***^ | 10.270^***^ |
|  | (0.109) | (0.089) | (0.216) | (0.113) | (0.145) | (0.084) |
| Age | 0.979^***^ | 0.987^***^ | 0.971^***^ | 0.979^***^ | 0.987^***^ | 0.970^***^ |
|  | (0.007) | (0.006) | (0.027) | (0.007) | (0.006) | (0.026) |
| Hukou | 0.712^***^ | 0.673^***^ | 1.647^**^ | 14.699^***^ | 48.650^***^ | 4.466^***^ |
|  | (0.050) | (0.082) | (0.694) | (0.526) | (0.555) | (0.610) |
| Income | 1.018^***^ | 1.177^***^ | 0.804^***^ | 1.021^***^ | 1.183^***^ | 0.814^***^ |
|  | (0.055) | (0.012) | (0.145) | (0.054) | (0.013) | (0.155) |
| Gender | 0.951^***^ | 0.993^***^ | 0.925^***^ | 0.949^***^ | 1.000^***^ | 0.921^***^ |
|  | (0.131) | (0.053) | (0.204) | (0.128) | (0.050) | (0.210) |
| Martial status | 0.772^*^ | 0.733^**^ | 3.443^***^ | 0.744^*^ | 0.705^**^ | 3.567^***^ |
|  | (0.396) | (0.317) | (0.324) | (0.401) | (0.317) | (0.295) |
| Education | 0.960^***^ | 1.075^***^ | 0.934^***^ | 0.957^***^ | 1.070^***^ | 0.914^***^ |
|  | (0.016) | (0.023) | (0.360) | (0.016) | (0.024) | (0.349) |
| Health insurance | 1.532^***^ | 1.236^***^ | 0.624 | 1.610^***^ | 1.349^***^ | 0.660 |
|  | (0.208) | (0.228) | (0.627) | (0.205) | (0.251) | (0.684) |
| PCC-6 * Residence |  |  |  | 0.507^***^ | 0.367^***^ | 0.768^***^ |
|  |  |  |  | (0.120) | (0.142) | (0.226) |
| Constant | 0.209 | 0.020 | 0.047 | 0.020 | 0.001 | 0.028 |
|  | (0.552) | (0.362) | (0.928) | (0.450) | (0.632) | (1.285) |
|  | | | | | | |
| Observations | 5,199 | 5,199 | 5,199 | 5,199 | 5,199 | 5,199 |
| Adj-R^2^ | 0.117 | 0.133 | 0.200 | 0.121 | 0.139 | 0.201 |
|  | | | | | | |
| Note: | ^*^p^**^p^***^p<0.01 | | | | | |
