# Supplementary material for: More patient-centered care, better healthcare: the association between patient-centered care and healthcare outcomes in inpatients
Source: Front Public Health. 2023 Oct 19;11:1148277. doi: 10.3389/fpubh.2023.1148277 (PMC10620693; doi:10.3389/fpubh.2023.1148277)
Supplement: Supplementary file 2 [file Table_2.DOCX]

|  | | | | |
| --- | --- | --- | --- | --- |
|  | Dependent variable: | | | |
|  |  | | | |
|  | ABM | PO | IU | RDR |
|  | (1) | (2) | (3) | (4) |
|  | | | | |
| PCC-6 | 0.356^***^ | 0.312^***^ | 0.238^***^ | 0.265^***^ |
|  | (0.089) | (0.084) | (0.031) | (0.049) |
| Age | 0.983^***^ | 0.957^***^ | 0.998^***^ | 1.007^***^ |
|  | (0.002) | (0.005) | (0.014) | (0.013) |
| Hukou | 1.010^***^ | 0.793^***^ | 0.620 | 0.590 |
|  | (0.176) | (0.257) | (0.405) | (0.411) |
| Income | 0.835^***^ | 0.923^***^ | 0.863^***^ | 0.862^***^ |
|  | (0.032) | (0.025) | (0.042) | (0.058) |
| Gender | 0.949^***^ | 0.734^***^ | 0.746^***^ | 0.648^***^ |
|  | (0.017) | (0.141) | (0.066) | (0.116) |
| Martial status | 1.544^***^ | 3.690^***^ | 0.477^***^ | 0.361^***^ |
|  | (0.231) | (0.233) | (0.182) | (0.089) |
| Education | 0.923^***^ | 0.967^***^ | 0.725^***^ | 0.745^***^ |
|  | (0.009) | (0.056) | (0.114) | (0.120) |
| Health insurance | 1.888^***^ | 1.863^***^ | 2.444^***^ | 2.845^***^ |
|  | (0.143) | (0.081) | (0.172) | (0.162) |
| Constant | 12.207^***^ | 21.747^***^ | 109.301^***^ | 48.909^***^ |
|  | (0.419) | (0.299) | (0.907) | (0.466) |
|  | | | | |
| Observations | 5,199 | 5,199 | 5,199 | 5,199 |
| Adj-R^2^ | 0.097 | 0.147 | 0.176 | 0.178 |
|  | | | | |
| Note: | ^*^p^**^p^***^p<0.01 | | | |
